# The Personalized Priority and Progress Questionnaire (PPPQ): A personalized instrument for quality of life and self-management for use in clinical trials and practice

**DOI:** 10.1007/s11136-023-03429-7

**Published:** 2023-05-12

**Authors:** Judith Tommel, Cinderella K. Cardol, Andrea W. M. Evers, Rianne Stuivenberg, Sandra van Dijk, Henriët van Middendorp

**Affiliations:** Health, Medical and Neuropsychology Unit, Institute of Psychology, Faculty of Social and Behavioural Sciences, Leiden University, Wassenaarseweg 52, 2333 AK Leiden, The Netherlands

**Keywords:** Personalized outcome, Patient-centered care, Patient priorities, Quality of life, Self-management, Chronic disease

## Abstract

**Purpose:**

The aim of this study was to develop and validate a brief personalized instrument that (1) defines patients’ priorities for improvement, (2) measures progress in prioritized quality of life (QoL) and self-management outcomes, and (3) is applicable in both clinical practice and clinical trials.

**Methods:**

The instrument was developed based on the literature on personalized assessment and patient priorities, feedback by clinicians, and six cognitive interviews with patients with chronic kidney disease. The resulting questionnaire, the Personalized Priority and Progress Questionnaire (PPPQ), contains a baseline and follow-op measurement. The baseline measurement assesses functioning on QoL (8 items) and self-management (5 items). The final item evaluates patients’ priorities for improvement. The follow-up measurement assesses progress in QoL and self-management. A personalized progress score can be calculated indicating the amount of progress on the QoL or self-management domain that is prioritized by the individual patient. Psychometric properties of the PPPQ were evaluated among patients with chronic kidney disease (*n* = 121) and patients with kidney failure treated with dialysis (*n* = 22).

**Results:**

The PPPQ showed to be a feasible instrument that is easy and quick to complete. Regarding the construct validity, small to large correlations were found between the items and existing validated questionnaires measuring related constructs.

**Conclusion:**

The PPPQ proved to be a feasible and valid instrument. The PPPQ can be adapted to match diverse populations and could be a useful tool both in clinical practice (e.g., to identify priorities and tailor treatment) and clinical trials (e.g., to evaluate the effectiveness of personalized interventions).

**Supplementary Information:**

The online version contains supplementary material available at 10.1007/s11136-023-03429-7.

## Plain English Summary

1. **What is the key problem?**
  Treating every patient with the same treatment would mean that every patient needs the same things. However, patients are not the same. Patients differ in health, needs, preferences, and personal situations. Therefore, it is better to include these personal differences and to make sure that the treatment is the right fit. A tool that aids patients in making clear what they find important about their health and life, can help to personalize healthcare.
2. **What is the main point of this study?**
  In this study, we developed a tool that supports patients in defining their priorities for improvement and measures changes in functioning. The tool consists of a personalized questionnaire that includes questions on quality of life (e.g., fatigue, pain, mood, social environment, daily activities) and self-management (e.g., diet, physical activity, smoking). In the final question, patients select the topics they find most important to improve on.
3. **What do the results mean?**
  The questionnaire showed to be a good, practical tool. In healthcare settings, patients could complete the questionnaire before every doctor’s appointment. In this way, doctors could keep track on patients’ functioning and use the results to discuss what patients need and what kind of treatment would fit. In research settings, researchers could use the results of the questionnaire to calculate how much patients’ functioning changed on the topics patients find most important. This is useful when evaluating whether a personalized treatment works.

## Introduction

Patients vary in functioning, preferences, goals, and values [1, 2]. Besides patients’ biological and clinical functioning, these individual differences and priorities should be incorporated in interventions [3–5]. Patients with chronic kidney disease (CKD) have expressed a need for holistic care that includes all aspects of a person’s health and wellbeing, including quality of life (QoL) and self-management behaviors (e.g., physical activity, dietary changes, medication use, non-smoking) [2, 6]. Moving away from a ‘mechanistic’ focus on laboratory results and focusing on patients’ actual wellbeing instead, is key for patient-centered care (PCC) [6]. PCC is defined as providing care that is respectful of and responsive to patient preferences, needs, and values and ensuring that patient values guide all clinical decisions [7]. Positive associations of PCC with enhanced QoL, wellbeing, patient satisfaction, perceived quality of care, and self-management [8–10], as well as improved clinical outcomes have been found, for example reductions in pain, blood pressure, complications, and hospitalization [9]. Thus, instead of evaluating one-size-fits-all interventions, the focus should be on identifying and offering the best intervention for *every individual patient* [11]. This calls for personalized (1) interventions and (2) outcome variables to do justice to each patient’s unique treatment trajectory [3, 4, 11].

As personalized interventions imply individual differences in treatment, standard generic outcome measures to evaluate their effectiveness will not suffice. Multiple questionnaires would be necessary to evaluate different treatment goals, which significantly harms the power of these studies since only the data of subgroups that worked on similar treatment goals can be used [4]. Moreover, generic measures invalidate the personalized character of the intervention by clouding patients’ results with unimportant or not focused-on health domains [3, 4, 11, 12]. Using personalized assessments enables to evaluate whether inventions are not only clinically, but also personally relevant to patients (i.e., personal utility) [13]. This allows general conclusions on treatment effectiveness, while considering each unique treatment trajectory. This feature makes personalized outcome measures highly valuable in research settings.

Personalized assessment can also be of great clinical value, as it helps to clarify patients’ needs and priorities. It provides a valuable asset in shared decision-making [12], in which patients have an active role in selecting treatment and care plans that match their preferences. Personalized assessments could also help to define personally-relevant treatment goals, which form the basis of personalized treatment [12]. Subsequently, personalized outcomes can monitor patient functioning over time [12]. Although incorporating patient priorities in decision-making and interventions is highly valued [7], they are not routinely assessed or recorded in medical records [14]. A personalized tool assessing priorities would make patient priorities explicitly visible in clinical practice.

However, adequate tools for use in clinical practice or trials are sparse. Several tools measure priorities or preferences, but all lack an effect assessment on health outcomes that are prioritized by patients themselves [14]. The McMaster Toronto Arthritis Patient Preference Disability Questionnaire (MACTAR) [15] assesses change in functioning areas that matter to patients, but limits feasibility by requiring trained interviewers and complex scoring [16]. Similarly, scales that focus on goal setting such as Goal Attainment Scaling (GAS) [17], the Patient Goal Priority Questionnaire (PGPQ) [18], and Self-Identified Goal Assessment (SIGA) [19] can help patients prioritize their needs for improvement, but are also time-consuming and require trained interviewers or therapists to help patients setting realistic goals [20].

This study aims to develop and validate a brief personalized instrument that is applicable to patients with diverse somatic conditions and that (1) defines patients’ priorities for improvement, (2) measures changes in functioning on patient-prioritized QoL and self-management outcomes, and (3) is applicable in both clinical practice and trials. This study evaluates the psychometric properties of the instrument in two different chronic kidney disease (CKD) samples. If feasible and valid, this brief, personalized tool can be used to easily identify, prioritize, and monitor individual problems and progress.

## Methods

### Study population

#### Questionnaire development

Using purposeful sampling, 4 patients with CKD and 2 patients with kidney failure treated with dialysis were recruited from Leiden University Medical Center to participate in the cognitive interviews.

#### Questionnaire evaluation

Datasets of two multicenter randomized controlled trials (RCT) were used, evaluating the effectiveness of a personalized e-health intervention in patients with CKD (E-GOAL study) [21] or kidney failure treated with dialysis (E-HELD study) [22].

Patient recruitment for the E-GOAL study took place from April 2018 through March 2020 within Dutch academic (Leiden University Medical Center, Leiden; Radboud university medical center, Nijmegen; University Medical Center Groningen, Groningen) and non-academic (Haaglanden Medical Center, The Hague) hospitals. Adult patients with CKD with an eGFR of 20–89 ml/min/1.73 m^2^ under treatment by an internist-nephrologist were invited to participate when their screening questionnaire indicated at least mild depressive or anxiety symptoms and that they failed to meet at least one of the nephrology self-management guidelines [23].

Patient recruitment for the E-HELD study took place from February 2019 through October 2021 within Dutch academic (Radboud university medical center, Nijmegen; Leiden University Medical Center, Leiden) and non-academic (VieCuri Medical Centre, Venlo; Bernhoven Hospital, Uden) hospitals and dialysis centers (Ravenstein Dialysis Centre, Ravenstein; Dialysis Center Groningen, Groningen). Adult patients with an eGFR < 15 ml/min/1.73 m^2^ treated with hemodialysis or peritoneal dialysis for at least three months were invited to participate when they presented low QoL or symptoms of fatigue, itch, depression, or anxiety on screening questionnaires.

Exclusion criteria for both studies were: age < 18 years; > 10% past-year renal-function loss, life expectancy < 12 months or serious psychiatric conditions, recent major stressful life events unrelated to kidney disease, study-interfering cognitive problems, psychological treatment, kidney transplant received or scheduled < 1 year ago or ahead, not fluent in Dutch language, pregnancy, and no access to a computer or internet. Additionally, the E-GOAL study excluded patients with anticipated need for dialysis work-up within the study time frame or a systolic blood pressure < 95 mmHg not responding to withdrawal or antihypertensives.

Ethical approval was obtained from the Medical Ethical Committee Leiden-Den Haag-Delft (E-GOAL: P17.172; E-HELD: P18.013).

### Item generation

Questionnaire topics were based on research team expertise and literature on frequently reported symptoms and patient priorities in the CKD and dialysis population [2, 24–27]. The questionnaire structure was based on existing personalized and goal-setting measurements [15–19]. Resulting items were judged on comprehensibility and relevance by medical psychologists and nephrologists and revised accordingly.

#### Cognitive interviews

Six cognitive interviews were conducted with patients to evaluate item feasibility, comprehensibility and readability. Additionally, patients were asked to judge the relevance of the QoL and self-management topics addressed by the items and were invited to add or remove items. The interviewers, JT and CKC, used the *think-aloud* approach and *verbal-probing techniques* to gain insight in patient response processes [28]. In this approach, the interviewees vocalize their thoughts while answering items. Verbal-probing techniques included paraphrasing and comprehension/interpretation, recall, specific, and general probes [28]. Based on the interviews, minor textual revisions were made to the questionnaire.

#### Personalized priority and progress questionnaire (PPPQ)

The resulting questionnaire, called Personalized Priority and Progress Questionnaire (PPPQ), consists of baseline and progress measurements:

*Baseline measurement:* assesses personal priorities for improvement in QoL areas and self-management behaviors.

- QoL: Whether patients experience limitations in QoL areas in the past 2 weeks is assessed by 8 items (fatigue, pain, itch, anxiety, depression, social environment, daily activities, and dependency), of which items can be omitted if not relevant in a particular population. Items are scored on a 5-point Likert scale (1 = not at all, 5 = extremely). An example item is “To what extent have you experienced limitations in the area of fatigue or sleep problems?”.
- Self-management: 5 items assess medication adherence, healthy diet, physical activity, weight maintenance, and non-smoking, using 5-point Likert scales (1 = not at all, 5 = extremely well). An example item is “To what extent have you managed to always take your medication as prescribed?”.
- Priorities: Patients make two top-2’s of the areas of QoL and self-management they prioritize for improvement and would actively commit to over the coming period.

*Progress measurement:* assesses progress in QoL or self-management behavior compared to baseline measurement.

- QoL and self-management: Patients indicate whether their experienced QoL limitations and self-management behaviors changed since the baseline measurement. The items are answered using a 7-point Likert scale (− 3 = many more, 0 = remained the same, + 3 = much fewer), following the base “Compared to the last time I completed this questionnaire, …”. Higher scores indicate improved QoL or self-management behavior. Example items are: “I now experience more/similar/fewer limitations in the area of fatigue or sleep problems” (QoL) and “I have managed less well/equally well/better to always take my medication as prescribed” (self-management).
- Priorities: Patients indicate whether they tried to improve in any QoL or self-management areas since the baseline measurement. Patients select a maximum of 2 QoL areas and 2 self-management behaviors or the options ‘other’ or ‘not applicable’ (when not worked on anything).

*Progress score.* The progress score indicates the amount of progress (i.e., change) on the QoL or self-management domain(s) prioritized by the patient. This score consists of the isolated scores on the progress items that were selected as priorities at baseline; if more than one was selected, an average progress score is calculated. Ultimately, this will result in one single score that includes all personally meaningful changes.

The original Dutch PPPQ was translated in English using the forward–backward method [29]. The English (PPPQ-EN) and Dutch (PPPQ-NL) versions are enclosed in Supplementary Files 1 and 2.

### Measures

#### Patient characteristics

Socio-demographic and clinical characteristics (age, sex, education level, marital status, and comorbidity) were collected using self-administered questionnaires.

#### Related constructs

An overview of the validated measures that were administered to evaluate the construct validity of the PPPQ can be found in Table 1. Higher scores indicate worse QoL, except for the energy, pain, social functioning, and daily activities measures. Regarding self-management behavior, higher scores indicate better self-management, except for smoking behavior and BMI.

**Table 1:** Measures administered to evaluate the construct validity of the Personalized Priority and Progress Questionnaire (PPPQ)

Construct	Measure
Quality of life areas	
Fatigue	Shortened Fatigue Questionnaire (SFQ) [48]
Sleeping problems	Sleep Problem Index II, Medical Outcomes Study (MOS) Sleep Scale [49]
Energy	Subscale Energy^a^, RAND Short Form-36 Health Status Inventory (RAND SF-36) [50]
Pain	Subscale Pain^a^, RAND Short Form-36 Health Status Inventory (RAND SF-36)) [50]
Itch	Impact of Chronic Skin Disease on Daily Life (ISDL) [51]
Anxiety	Generalized Anxiety Disorder 7-item Scale (GAD-7) [52]
Worrying	Penn State Worry Questionnaire (PSWQ) [53]
Depression	Patient Health Questionnaire depression scale (PHQ-9) [54]
Social functioning	Subscale Social functioning^a^, RAND Short Form-36 Health Status Inventory (RAND SF-36)) [50]
Perceived emotional support	Subscale Perceived support, Inventory for Social Reliance (ISR) [55]
Actual emotional support	Subscale Actual support, Inventory for Social Reliance (ISR) [55]
Mutual visiting	Mutual visiting, Inventory for Social Reliance (ISR) [55]
Daily activities	Subscale role limitations due to physical problems^a^, RAND Short Form-36 Health Status Inventory (RAND SF-36)) [50]
Self-management behaviors	
Self-management	Partners in Health Scale (PiH) [56]
Medication adherence	Simplified Medication Adherence Questionnaire (SMAQ) [57]
Dietary adherence	2 self-constructed questions^b^: “In the past week, how often have you kept a healthy diet?” (scored 1–5 from “never” to “always”) “In the past week, how well do you believe you have kept a healthy diet?” (score 1˗10 from “very badly” to “very well”)
Physical activity, hrs. per week	Short Questionnaire to Assess Health-enhancing physical activity (SQUASH) [58]
Physical activity, days per week minimally 30 min. active	Short Questionnaire to Assess Health-enhancing physical activity (SQUASH) [58]
Weight maintenance	Body mass index (BMI) [59], ratio of body weight (kg) and the square of height (m)
Smoking behavior	Current smoking on daily or nondaily basis) (yes/no) Amount of tobacco per day

^a^Scored as *T*-scores (Hays norm-based scoring algorithm)[50]

^b^The items were combined by a categorical principal-components analysis [60] to obtain a single *z*-score for dietary adherence

### Statistical analyses

The PPPQ does not intend to measure a single underlying concept, thus, homogeneity of items is not assumed. Consequently, no factor analysis was performed. The internal consistency and intercorrelations of the items were explored for possible associations [30, 31].

Descriptives were calculated of patient characteristics, PPPQ, and related constructs.

Item characteristics of the PPPQ were examined by evaluating the presence of floor and ceiling effects, based on more than 15% of patients achieving the lowest or highest possible score [30].

To examine construct validity, correlations were calculated between baseline PPPQ items and similar constructs. Additionally, since the progress scores assess change in QoL or self-management behavior, they were correlated with change scores of related constructs (follow-up subtracted by baseline). Due to the small number of patients in the E-HELD study, more emphasis was placed on the magnitude of the association than on statistical significance.

For the CKD sample, the QoL-item on dependency was not relevant and therefore not included. For the dialysis sample, self-management was not assessed, as it was not part of the intervention.

## Results

### Questionnaire development

#### Feasibility PPPQ

None of the interviewed patients reported difficulties with comprehending or answering the questionnaire: questions were considered clear and easy to understand. The QoL and self-management areas of were considered relevant for patients with a kidney disease. Additionally, patients had no trouble selecting areas as personal priorities. Patients completed the questionnaire in 2–4 min.

### Questionnaire evaluation

#### Patient characteristics

For the E-GOAL study, 460 patients with CKD not on dialysis completed screening questionnaires of whom 146 were eligible for randomization. Of these patients, 121 were included in the trial.

For the E-HELD study, 59 patients with kidney failure treated with dialysis completed screening questionnaires of whom 46 were eligible for randomization. Thirty-five were included in the trial, of whom 22 completed assessments needed for the current study’s analyses. Patient characteristics are shown in Table 2.

**Table 2:** Patient characteristics

	CKD patients (*N* = 121)	Dialysis patients (*N* = 22)
Age, mean (*SD*)	55.95 (13.87)	65.50 (11.68)
Median	57.31	67.00
Range	25.77–81.59	46.00–83.00
Male sex	56.7%	54.5%
Education level		
Lower	52.9%	45.5%
Higher	46.3%	54.5%
Unknown	0.8%	-
Marital status, with partner	73.6%	95.5%
Comorbidity	69.4%	86.4%
Hypertension	39.7%	50.0%
Heart disease	19.0%	40.9%
Diabetes	16.5%	31.8%
Gastrointestinal disease	9.1%	27.3%
Lung disease	6.6%	18.2%
Cancer	5.8%	9.1%
Physical quality of life (PCS, RAND SF-36)	35.97 (8.64)	34.18 (7.14)
Mental quality of life (MCS, RAND SF-36)	39.81 (8.68)	44.18 (9.22)

Lower education includes primary, pre-vocational, and vocational education; higher education includes advanced secondary and tertiary education.

*CKD* chronic kidney disease, *SD* standard deviation, *PCS* physical component summary, *RAND SF-36* RAND Short Form-36 Health Status Inventory, *MCS* mental component summary

### Descriptives PPPQ and related constructs

Table 3 provides the descriptives of the PPPQ and related constructs. Internal consistency of the baseline PPPQ QoL items was *α* = 0.74 (CKD sample) and *α* = 0.60 (dialysis sample) and of the progress items *α* = 0.88 (CKD sample) and *α* = 0.80 (dialysis sample). In the CKD sample, the self-management items showed an internal consistency of *α* = 0.42 (baseline items) and *α* = 0.69 (progress items). Intercorrelations between the PPPQ items can be found in Supplementary File 3.

**Table 3:** Descriptives of the Personalized Priority and Progress Questionnaire (PPPQ) and measures of related constructs

	CKD patients (*N* = 121)				Dialysis patients (*N* = 22)			
	Baseline		Follow-up^a^		Baseline		Follow-up^b^	
	Mean (*SD*)	α	Mean (*SD*)	Α	Mean (*SD*)	α	Mean (*SD*)	α
PPPQ QoL items^c^	2.15 (0.63)	.74	0.42 (0.91)	.88	1.94 (0.43)	.60	0.27 (0.82)	.80
PPPQ self-management items^c^	3.56 (0.58)	.42	0.28 (0.74)	.69	–	–	–	–
Fatigue (SFQ)	18.98 (5.16)	.85	17.10 (5.94)	.89	20.59 (5.47)	.87	19.54 (5.75)	.86
Sleeping problems (Sleep Problem Index II, MOS Sleep scale)	40.63 (15.89)	.81	36.16 (15.90)	.81	–	–	–	–
Energy (Energy scale, RAND SF-36)	42.29 (6.41)	.63	44.31 (7.70)	.76	44.77 (8.92)	.82	41.91 (7.19)	.67
Pain (subscale RAND SF-36)	44.26 (9.60)	.73	46.33 (9.82)	.74	47.64 (11.17)	.76	47.23 (10.16)	.66
Itch (ISDL)	–	–	–	–	7.05 (2.89)	.88	6.87 (2.44)	.80
Anxiety (GAD-7)	5.49 (3.78)	.81	3.95 (3.16)	.81	2.36 (1.92)	.68	2.55 (2.44)	.72
Worrying (PSWQ)	44.79 (10.94)	.91	42.01 (11.33)	.91	–	–	–	–
Depression (PHQ-9)	7.91 (3.33)	.54	5.76 (3.78)	.74	5.73 (3.67)	.77	7.14 (4.39)	.79
Social functioning (subscale RAND SF-36)	39.07 (9.67)	.68	42.24 (10.50)	.79	39.68 (9.78)	.56	35.68 (10.47)	.78
Perceived emotional support (ISR)	14.47 (3.52)	.82	14.89 (3.47)	.82	15.68 (3.71)	.81	15.82 (3.58)	.88
Actual emotional support (ISR)	7.25 (2.00)	.77	7.22 (1.84)	.72	6.36 (2.24)	.81	6.05 (2.01)	.80
Mutual visiting (ISR)	5.35 (1.42)	.74	5.35 (1.26)	.64	5.18 (1.53)	.84	4.81 (1.62)	.53
Role limitations due to physical problems (subscale RAND SF-36)	36.18 (11.44)	.82	40.77 (11.80)	.81	30.73 (9.40)	.88	33.05 (10.40)	.84
Self-management (PiH)	80.04 (9.63)	.78	82.74 (9.27)	.81	–	–	–	–
Medication adherence (SMAQ)	5.16 (1.05)	n/a	5.25 (0.95)	n/a	–	–	–	–
Dietary adherence	0.00 (0.93)	n/a	0.35 (0.76)	n/a	–	–	–	–
Physical activity, hrs. per week (SQUASH)	17.10 (16.04)	n/a	15.54 (15.52)	n/a	–	–	–	–
BMI	27.38 (5.33)	n/a	27.16 (5.35)	n/a	–	–	–	–
Smoking, %	9.1	n/a	8.3	n/a	–	–	–	–
Amount of tobacco per day, units	0.80 (3.31)	n/a	0.50 (2.75)	n/a	–	–	–	–

Mean and standard deviations shown as mean (*SD*); internal consistency: Cronbach’s α; Follow-up^a^: 3 months follow-up; Follow-up^b^: 6 months follow-up; PPPQ QoL/self-management items^c^: average sum scores, items differ per assessment point, i.e. the baseline measurement assessed baseline items and the follow-up measurement assessed progress items

*CKD* chronic kidney disease, *SD* standard deviation; *QoL* quality of life, *SFQ* Shortened Fatigue Questionnaire (SFQ), *MOS Sleep Scale* Medical Outcomes Study Sleep Scale, *RAND SF-36* RAND Short Form-36 Health Status Inventory, *ISDL* Impact of Chronic Skin Disease on Daily Life, *GAD-7* Generalized Anxiety Disorder 7-item Scale, *PSWQ* Penn State Worry Questionnaire, *PHQ-9* Patient Health Questionnaire depression scale, *ISR* Inventory for Social Reliance, *PiH* Partners in Health Scale, *SMAQ* Simplified Medication Adherence Questionnaire, *SQUASH* Short Questionnaire to Assess Health-enhancing physical activity, *BMI* body mass index

### Item characteristics PPPQ

#### QoL

Item characteristics of the QoL items are shown in Table 4. At baseline, mean item scores ranged from 1.61 (itch) to 3.15 (fatigue) in the CKD sample and from 1.55 (anxiety and depression) to 2.86 (fatigue) in the dialysis sample. Most items covered most of the 1–5 range, except for pain and depression (1–3), and anxiety (1–2) in the dialysis sample. Floor effects were found for all items except fatigue. No ceiling effects were detected.

**Table 4:** Characteristics of the Personalized Priority and Progress Questionnaire (PPPQ) items

	Baseline								Follow-up							
	CKD patients (*N* = 121)				Dialysis patients (*N* = 22)				CKD patients (*N* = 121)				Dialysis patients (*N* = 22)			
Item	Mean (*SD*)	Floor (%)	Ceiling (%)	Range	Mean (*SD*)	Floor (%)	Ceiling (%)	Range	Mean (*SD*)	Floor (%)	Ceiling (%)	Range	Mean (*SD*)	Floor (%)	Ceiling (%)	Range
QoL items																
Fatigue	3.15 (1.12)	5.0	13.2	1–5	2.86 (1.04)	4.5	9.1	1–5	0.21 (1.14)	0.8	2.5	− 3 to 3	− 0.45 (1.18)	4.5	0	− 3 to 2
Pain	2.08 (1.05)	36.4	0.8	1–5	1.82 (0.85)	45.5	0	1–3	0.32 (1.21)	1.7	8.3	− 3 to 3	0.27 (1.07)	0	4.5	− 2 to 3
Itch	1.61 (0.93)	62.0	0.8	1–5	2.05 (0.72)	18.2	0	1–4	0.56 (1.25)	0	13.2	− 2 to 3	0.45 (1.10)	0	4.5	− 1 to 3
Anxiety	2.02 (0.91)	30.6	0.8	1–5	1.55 (0.51)	45.5	0	1–2	0.57 (1.25)	0.8	8.3	− 3 to 3	0.59 (1.44)	0	18.2	− 2 to 3
Depression	1.79 (0.87)	43.8	0	1–4	1.55 (0.60)	50.0	0	1–3	0.62 (1.29)	0.8	9.9	− 3 to 3	0.59 (1.47)	0	18.2	− 2 to 3
Social environment	1.90 (0.96)	39.7	3.3	1–5	1.59 (0.96)	63.6	0	1–4	0.33 (1.15)	0.8	5.8	− 3 to 3	0.41 (1.30)	0	13.6	− 2 to 3
Daily activities	2.50 (1.14)	19.8	5.8	1–5	2.27 (0.99)	18.2	0	1–4	0.32 (1.14)	1.7	5.0	− 3 to 3	0.36 (1.09)	0	4.5	− 1 to 3
Dependency	–	–	–	–	1.86 (0.89)	40.9	0	1–4	–	–	–	–	− 0.09 (1.34)	4.5	4.5	− 3 to 3
Self-management items																
Medication adherence	4.45 (0.77)	0	58.7	2–5	–	–	–	–	0.23 (0.78)	0	4.1	− 2 to 3	–	–	–	–
Healthy diet	3.39 (0.90)	1.7	8.3	1–5	–	–	–	–	0.29 (1.04)	0.8	3.3	− 3 to 3	–	–	–	–
Physical activity	2.82 (1.04)	9.9	4.1	1–5	–	–	–	–	0.29 (1.21)	0	5.8	− 2 to 3	–	–	–	–
Weight maintenance	2.64 (1.23)	20.7	7.4	1–5	–	–	–	–	0.17 (1.16)	0	4.1	− 2 to 3	–	–	–	–
Non-smoking	4.52 (1.24)	9.9	85.1	1–5	–	–	–	–	0.45 (1.29)	2.5	14.9	− 3 to 3	–	–	–	–

Follow-up measures for the CKD and dialysis population took place at, respectively, 3 and 6 months follow-up.

*CKD* chronic kidney disease, *SD* standard deviation, *QoL* quality of life

Regarding the progress items, mean item scores ranged from 0.21 (fatigue) to 0.62 (depression) in the CKD sample and from -0.45 (fatigue) to 0.59 (anxiety and depression) in the dialysis sample. All items covered a range of at least 4 out of 7 (− 3 to 3). No floor effects were detected and only in the dialysis sample ceiling effects were detected for anxiety and depression.

### Self-management

Item characteristics of the self-management items are shown in Table 4. Mean baseline item scores ranged from 2.64 (weight maintenance) to 4.52 (non-smoking) and mean progress scores from 0.17 (weight maintenance) to 0.45 (non-smoking). All items covered at least 4 out of 5 baseline scores and at least 6 out of 7 progress scores. A floor effect was found for baseline weight maintenance. Ceiling effects were found for baseline medication adherence and non-smoking. No floor and ceiling effects were detected for the progress items.

### Construct validity PPPQ

#### QoL

Results of the baseline and progress QoL items can be found in Table 5 and 6, respectively. Across samples, most baseline items correlated at least moderately with related constructs [(−)0.31 ≤ *r* ≤ 0.70], except for non-meaningful or small correlations of social environment with emotional support and mutual visiting and, in the dialysis sample, of daily activities with role limitations due to physical problems and of dependency with emotional support and mutual visiting (*r*-values ≤ − 0.22).

**Table 5:** Construct validity of the baseline quality of life items of the Personalized Priority and Progress Questionnaire (PPPQ) (Pearson correlations)

		Fatigue	Pain	Itch	Anxiety	Depression	Social environment	Daily activities	Dependency
Fatigue (SFQ)	CKD: Dialysis:	.47** .41	–	–	–	–	–	–	–
Sleeping problems (Sleep Problem Index II, MOS Sleep scale)	CKD: Dialysis:	.42**	–	–	–	–	–	–	–
Energy (subscale, RAND SF-36)	CKD: Dialysis:	− .42** − .43*	–	–	–	–	–	–	–
Pain (subscale RAND SF-36)	CKD: Dialysis:	–	–.68** − .70*	–	–	–	–	–	–
Itch (ISDL)	CKD: Dialysis:	–	–	– .68**	–	–	–	–	–
Anxiety (GAD-7)	CKD: Dialysis:	–	–	–	.67** .32	-	–	–	–
Worrying (PSWQ)	CKD: Dialysis:	–	–	–	.55**	-	–	–	–
Depression (PHQ-9)	CKD: Dialysis:	–	–	–	–	.46** .70**	–	–	–
Social functioning (subscale RAND SF-36)	CKD: Dialysis:	–	–	–	–	–	− .38** − .44**	–	– − .35
Perceived emotional support (ISR)	CKD: Dialysis:	–	–	–	–	–	-.09 -.31	–	– .13
Actual emotional support (ISR)	CKD: Dialysis:	–	–	–	–	–	-.02 − .22	–	– .002
Mutual visiting (ISR)	CKD: Dialysis:	–	–	–	–	–	− .10 − .21	–	– − .16
Role limitations due to physical problems (subscale RAND SF-36)	CKD: Dialysis:	–	–	–	–	–	–	− .42** − .003	–

*CKD* chronic kidney disease, *SFQ* Shortened Fatigue Questionnaire (SFQ), *MOS Sleep Scale* Medical Outcomes Study Sleep Scale, *RAND SF-36* RAND Short Form-36 Health Status Inventory, *ISDL* Impact of Chronic Skin Disease on Daily Life, *GAD-7* Generalized Anxiety Disorder 7-item Scale, *PSWQ* Penn State Worry Questionnaire, *PHQ-9* Patient Health Questionnaire depression scale, *ISR* Inventory for Social Reliance

*p < *.*05; **p < *.*01; CKD patients (*N* = 121); Dialysis patients (*N* = 22)

**Table 6:** Construct validity of the quality of life items of the Personalized Priority and Progress Questionnaire (PPPQ) and change scores of measurements assessing related constructs (Pearson correlations)

		Fatigue	Pain	Itch	Anxiety	Depression	Social environment	Daily activities	Dependency
Fatigue (SFQ)	CKD: Dialysis:	− .36** − .39	–	–	–	–	–	–	–
Sleeping problems (Sleep Problem Index II, MOS Sleep scale)	CKD: Dialysis:	− .30**	–	–	–	–	–	–	–
Energy (subscale, RAND SF-36)	CKD: Dialysis:	.36** .39	–	–	–	–	–	–	-
Pain (subscale RAND SF-36)	CKD: Dialysis:	–	.14 .05	–	–	–	–	–	–
Itch (IHDL)	CKD: Dialysis:	–	–	– − .43*	-	–	–	–	–
Anxiety (GAD-7)	CKD: Dialysis:	–	-	-	− .21* − .15	–	–	–	–
Worrying (PSWQ)	CKD: Dialysis:	–	-	-	− .11 –	–	–	–	–
Depression (PHQ-9)	CKD: Dialysis:	–	-	-	–	− .35** − .04	–	–	–
Social functioning (subscale RAND SF-36)	CKD: Dialysis:	–	–	–	–	-	.14 .13	–	− .30
Perceived emotional support (ISR)	CKD: Dialysis:	–	–	–	–	–	.03 − .41	–	− .27
Actual emotional support (ISR)	CKD: Dialysis:	–	–	–	–	–	− .03 − .20	–	– − .32
Mutual visiting (ISR)	CKD: Dialysis:	–	–	–	–	–	.004 .20	–	− .30
Role limitations due to physical problems (subscale RAND SF-36)	CKD: Dialysis:	–	–	–	–	–	–	.21* − .06	–

*CKD* chronic kidney disease, *SFQ* Shortened Fatigue Questionnaire (SFQ), *MOS Sleep Scale* Medical Outcomes Study Sleep Scale, *RAND SF-36* RAND Short Form-36 Health Status Inventory, *ISDL* Impact of Chronic Skin Disease on Daily Life, *GAD-7* Generalized Anxiety Disorder 7-item Scale, *PSWQ* Penn State Worry Questionnaire, *PHQ-9* Patient Health Questionnaire depression scale, *ISR* Inventory for Social Reliance

*p < *.*05; **p < *.*01; CKD patients (*N* = 121); Dialysis patients (*N* = 22)

Across samples, at least moderate correlations were found of the QoL progress scores with changes in related constructs [0.30 ≤ *r* ≤ (−)0.43], except for pain, anxiety, social environment, and daily activities with change in their related construct and, in the dialysis sample, for depression and dependency (*r*-values ≤  0.27).

### Self-management

Results of the baseline and progress self-management items can be found in Tables 7 and 8, respectively. Most items showed at least moderate correlations with related constructs [0.43 ≤ *r* ≤ (−)0.66], except for physical activity (*r* = 0.23) and self-management (*r*-values ≤ 0.27).

**Table 7:** Construct validity of the baseline self-management items of the Personalized Priority and Progress Questionnaire (PPPQ) (Pearson correlation) in a CKD sample (Pearson correlation)

	Medication adherence	Healthy diet	Physical activity	Weight maintenance	Non-smoking
Self-management (PiH)	.19*	.27**	.23*	.22*	.25**
Medication adherence (SMAQ)	.44**	–	–	–	–
Dietary adherence	–	.61**	–	–	–
Physical activity, hrs. per week (SQUASH)	–	–	.23*	-	–
Physical activity, days per week minimally 30 min. active			.43**		
BMI	–	–	–	− .63**	–
Amount of tobacco per day					− .66**

*CKD* chronic kidney disease, *PiH* Partners in Health Scale, *SMAQ* Simplified Medication Adherence Questionnaire, *SQUASH* Short Questionnaire to Assess Health-enhancing physical activity, *BMI* body mass index

*p < *.*05; **p < *.*01, *N* = 121, CKD patients

**Table 8:** Construct validity of self-management progress items of the Personalized Priority and Progress Questionnaire (PPPQ) and change scores of measurements assessing similar constructs (Pearson correlations) in a CKD sample

	Medication adherence	Healthy diet	Physical activity	Weight maintenance	Non-smoking
Self-management (PiH)	.09	.26**	.40**	.31**	−.02
Medication adherence (SMAQ)	.15	–	–	–	-
Dietary adherence	–	.29**	–	–	–
Physical activity, hrs. per week (SQUASH)	–	-	−.12	–	–
Physical activity, days per week minimally 30 min. active			.18		
BMI	–	-	–	−.18	–
Amount of tobacco per day					−.25**

Change scores of the measurements assessing similar constructs were calculated by subtracting mean scores at baseline from mean scores at follow-up

*CKD* chronic kidney disease, *PiH* Partners in Health Scale, *SMAQ* Simplified Medication Adherence Questionnaire, *SQUASH* Short Questionnaire to Assess Health-enhancing physical activity, *BMI* body mass index

^**^p < *.*01, *N* = 121, CKD patients

The progress in physical activity (*r* = 0.40) and weight maintenance (*r* = 0.31) showed moderate correlations with change in self-management. The other items showed small correlations with their related constructs and change in self-management (*r*-values ≤ 0.29).

## Discussion

This study aimed to develop a brief personalized instrument that (1) defines patients’ priorities for improvement, (2) measures change in QoL and self-management outcomes prioritized by individual patients, and (3) is applicable in both clinical practice and clinical trials. The resulting questionnaire, the PPPQ, includes baseline and follow-up measurements. The baseline measurement assesses personal QoL and self-management priorities for improvement. The follow-up measurement assesses the amount of self-perceived progress in QoL or self-management compared to baseline. A progress score indicates the amount of progress on the QoL or self-management area(s) prioritized by the individual patient. The PPPQ was evaluated in two kidney disease samples, showing to be a valid and feasible instrument that is easy and quick to complete.

The PPPQ showed to have good construct validity. Regarding the baseline items, moderate to large correlations were found between all items and validated questionnaires measuring related constructs. Only the scales measuring emotional support and mutual visiting did not show significant correlations with the PPPQ items social functioning and dependency. Possibly, social support was a too different construct not necessarily related to social functioning and dependency [32, 33].

Correlations between the progress items and related constructs were somewhat smaller than the correlations of the baseline items. Possibly, this is the result of different ways of determining progress. In the PPPQ, patients make their own comparison of their current versus their previous functioning, while for the related constructs change was based on the difference between two different assessments. Possibly, patients’ self-perceived change in functioning is influenced by ‘response shift’, including change in internal standards and values as part of disease adaptation [34]. No correlations were found between anxiety and depression progress and the anxiety and depression scales in the dialysis sample, which could result from almost none of the dialysis patients reporting anxiety or depression symptoms (floor effects) [30]. In the CKD patients, where at least mild symptoms of anxiety or depression were inclusion criteria, significant associations were found. To conclude, evidence was found for good construct validity of the baseline measurement, with less determined outcomes for the progress items.

Despite the PPPQ including several domains of QoL and self-management, the internal consistency of the QoL items was surprisingly good. The self-management items showed lower internal consistency, possibly because they are less related in terms of content. Weight maintenance, for example, is not necessarily related to medication adherence.

Several floor and ceiling effects were detected for the baseline items. Particularly in the dialysis sample, the small sample size probably resulted in a smaller response range. Normally, floor and ceiling effects would decrease the responsiveness of a questionnaire: they make it difficult to detect an intervention effect in participants who score on the lower scale levels before an intervention [35]. Since the PPPQ is a personalized scale that specifically addresses changes in areas patients find important, this will not be a problem. Besides, the progress score is based on the progress items that rarely showed floor or ceiling effects.

## Strengths and limitations

It is increasingly recognized that a one-size-fits all approach to healthcare falls short to the complexity and diversity of individual patients. Shifting to patient-centered care (PCC) helps to better understand individual patient needs [8–10, 36]. For PCC to succeed, adequate tools that promote personalization are required [4]. While current instruments lack the possibility to assess an effect on outcomes that are valued by patients [14], the PPPQ identifies personally meaningful areas and uses these progress scores as an outcome measure, making it a valuable PCC tool. Another strength is the possibility to apply the PPPQ in diverse populations. The self-management items capture a range of generic lifestyle behaviors that are important for chronic disease management and prioritized by patients with chronic disease [23, 37]. The QoL items include issues that are relevant in many chronic conditions. For example, symptoms such as fatigue, sleep problems, and pain, and the impact of disease on patients’ emotional wellbeing, daily and social life are also prioritized by people with rheumatoid arthritis [38], COPD, asthma [39], spinal cord injury [40], breast cancer [41], and lung cancer [42]. Additionally, the PPPQ is flexible when it comes to removing possibly irrelevant items (e.g., itch) or adding items that are a specific problem in a particular population (e.g., dependency in dialysis patients) [2]. Depending on the relevance, it is also possible to only administer the QoL or self-management items. In this way the PPPQ can be adapted to match the specific needs of a population. Another strength is the ease and speed in which this questionnaire can be completed by patients. This is a great advantage compared to existing personalized instruments that are usually time-consuming and require trained interviewers or therapists [16].

A limitation of this study is the relatively small sample size, especially regarding the dialysis sample. Therefore, we focused more on the magnitude than on the significance of the associations. For a more robust examination of the validity of the PPPQ, larger samples of patients with diverse medical conditions are advised. Research among patients with conditions other than CKD or kidney failure is also needed to determine the applicability of the PPPQ in these populations. Another limitation is the lack of a gold standard that measures personalized health outcomes that are prioritized by patients [20]. Consequently, we had to select different questionnaires for each item to evaluate the construct validity.

## Implications

The PPPQ could be of use in both clinical and research settings. Box 1 provides an overview of the applicability. In clinical settings, the PPPQ could be used as a brief tool to evaluate patients’ priorities and to keep track of patients’ functioning. By this, the PPPQ could be used to evaluate patients’ functioning in general, similar to, but shorter than, QoL questionnaires, to specifically zoom in on QoL and self-management areas that patients themselves find important. The PPPQ could be completed on a routine basis and the results discussed during patient-clinician consultations. In this way, the PPPQ results can form the starting point of a discussion on patient priorities and shared decision-making to decide on a personalized treatment plan. Patients usually find it difficult to discuss their priorities, especially if not explicitly asked by clinicians [43, 44], and clinicians may find it difficult to know what to ask each patient and lack time to discuss all potential QoL areas or self-management behaviors. The PPPQ could make it easier for patients to discuss their particular difficulties and needs. Thereby, patient-clinician communication can be facilitated [45, 46].

**Box 1:** Implications of the Personalized Priority and Progress Questionnaire (PPPQ) in clinical and research settings

Clinical settings	Research settings
• Identify patient priorities	• Evaluate personalized interventions by using the progress score
• Use as conversation starter for a talk on patient priorities and patient needs	• Add or remove items to match the specific needs of the study population
• Use to support shared decision-making and tailor treatment based on results	• Use both the QoL and self-management items or only the QoL or self-management items to match the specific research questions
• Monitor patients’ QoL and adherence to self-management behaviors	• All implications listed under clinical settings are applicable in intervention studies as well

QoL, quality of life

In research settings, the PPPQ is an ideal tool to evaluate the effectiveness of personalized interventions, where treatment goals vary per participant. Some participants may work on improving their coping skills regarding fatigue, while others work on improving their social relationships. When evaluating personalized treatments using general health outcomes, outcomes will be clouded by scores on areas that may be unimportant to patients and, therefore, the personalized character will be lost [3, 4, 11, 12]. Additionally, multiple questionnaires would be necessary to evaluate different treatment goals (e.g., questionnaires on fatigue and social relationships), with the consequence of decreased power, since only part of the participants worked on those particular areas [4]. Ideally, researchers would have one overall score that justifies the personalized character of the intervention. We believe the PPPQ progress score could be that score. With this score, scores on personally meaningful areas are isolated, resulting in one single score that researchers can analyze across participants. When determining this progress score, researchers can use the priorities as selected at baseline or the areas patients indicated to have actively worked on at follow-up. The latter option can be useful if there is indication of switched treatment goals over the study course. Additionally, this option can be used as check question to find out whether patients in the control condition spontaneously worked on their health. For trials with waiting-list or care-as-usual control conditions, we advise to use the baseline-selected priorities. This is in line with existing personalized measurements such as MACTAR and GAS [15, 16] that advice patients to set goals prior to randomization, which enables researchers to apply the same calculations to both control and intervention conditions [15, 16, 47].

## Conclusion

To identify and monitor patient priorities over time, the PPPQ was developed. The PPPQ can be used in both clinical and research settings and proved to be a valid questionnaire that patients can easily complete without needing assistance. The PPPQ is a personalized scale that addresses changes in areas prioritized by patients themselves. A progress score can be calculated based on the areas that are personally meaningful to the individual patient. This great benefit makes the PPPQ a suitable instrument to evaluate personalized interventions in which patients work on different treatment goals. In clinical settings, the PPPQ could be used as a quick and easy tool to evaluate patients’ priorities and to monitor their functioning. With these characteristics, the PPPQ could aid in delivering high-quality care, tailored to the unique needs and priorities of every individual patient.

## Supplementary Information

Below is the link to the electronic supplementary material.

Supplementary file 1: English version PPPQ (PDF 204 KB)

Supplementary file 2: Dutch version PPPQ (PDF 199 KB)

Supplementary file 3: Intercorrelations PPPQ items (PDF 135 KB)

## Data Availability

The pseudonymized datasets and syntaxes can be made available by the corresponding author upon reasonable request.
