# Supplementary material for: The Personalized Priority and Progress Questionnaire (PPPQ): A personalized instrument for quality of life and self-management for use in clinical trials and practice
Source: Qual Life Res. 2023 May 12;32(10):2789–803. doi: 10.1007/s11136-023-03429-7 (PMC10474184; doi:10.1007/s11136-023-03429-7)
Supplement: Supplementary file 1 — English version PPPQ (PDF 204 KB) [file 11136_2023_3429_MOESM1_ESM.pdf]

Personalized Priority and Progress Questionnaire (PPPQ)

Quality of life

1. Baseline: Score for current functioning – general

The following questions are about limitations that people may experience **due to their kidney disease**. Think about the past 2 weeks. Please indicate to what extent you have experienced **limitations in the following areas**.

GENERAL<br>To what extent have you experienced limitations in the area of…	*Not at all*	*Slightly*	*Moderately*	*Considerably*	*Extremely*
a. ...fatigue or sleep problems?	1	2	3	4	5
b. ...pain?	1	2	3	4	5
c. ...itching?	1	2	3	4	5
d. ...tension, anxiety, or worrying?	1	2	3	4	5
e. ...low mood or feeling down?	1	2	3	4	5
f. ...your social environment (e.g., communication about your needs or wishes, asking for or receiving support)?	1	2	3	4	5
g. ...daily activities (e.g., work, hobbies, or social activities)?	1	2	3	4	5
h. ...dependence on others?	1	2	3	4	5

2. Baseline: Setting priorities - general

Choose the top two areas related to your perceived limitations that you would **most like** to improve, and that you plan to work on actively over the coming period. Rank these in order of relevance, where priority 1 is the most relevant, and priority 2 the next most relevant.

Limitations in the area of...

1. fatigue or sleep problems
2. pain
3. itching
4. tension, anxiety, or worrying
5. low mood or feeling down
6. social environment
7. daily activities
8. dependence
9. other (please state):

a. Priority 1: ____________________

b. Priority 2: ____________________

3. Follow-up measurement: Score for self-perceived progress – general

The following questions are about limitations that people may experience **due to their kidney disease**. For the statements below, please indicate whether, compared to the last time you completed these questionnaires, you now experience more or fewer **limitations**, or whether your situation has remained the same. It is not a problem if you do not remember exactly what you entered the last time: a rough estimate will do.

GENERAL<br>Compared to the last time I completed this questionnaire, I now experience more/fewer limitations in the area of...	*Many more*	*More*	*Slightly more*	*Remained the same*	*Slightly fewer*	*Fewer*	*Much fewer*
a. fatigue or sleep problems.	-3	-2	-1	0	1	2	3
b. pain.	-3	-2	-1	0	1	2	3
c. itching.	-3	-2	-1	0	1	2	3
d. tension, anxiety, or worrying.	-3	-2	-1	0	1	2	3
e. low mood or feeling down.	-3	-2	-1	0	1	2	3
f. social environment (e.g., communication about your needs or wishes, asking for or receiving support).	-3	-2	-1	0	1	2	3
g. daily activities (e.g., work, hobbies, or social activities).	-3	-2	-1	0	1	2	3
h. dependence on others.	-3	-2	-1	0	1	2	3

4. Follow-up measurement: Areas actively worked on – general

Have you tried to improve anything in any of these areas over the recent period?

If so, please choose up to two areas below that you have actively tried to improve recently. Rank these two areas, where number 1 is the area you have worked on most, and number 2 the area you have worked on slightly less.

If you have not actively worked on any of these areas, choose "not applicable".

Limitations relating to..

1. fatigue or sleep problems
2. pain
3. itching
4. tension, anxiety, or worrying
5. low mood or feeling down
6. social environment
7. daily activities
8. dependence
9. other (please state):
10. not applicable

Area 1: I worked on limitations in the area of ____________________

Area 2: I worked on limitations in the area of ____________________

Self-management

1. **Baseline: Score for current functioning - self-management**

   The following questions are about self-management. Think about the past 2 weeks. Please indicate to what extent you have successfully managed to maintain a healthy lifestyle in the following areas.

SELF-MANAGEMENT<br>To what extent have you managed...	*Not at all*	*Slightly*	*Reasonably well*	*Well*	*Extremely well*
a. ...to always take your medication as prescribed?	1	2	3	4	5
b. ...to eat healthily?	1	2	3	4	5
c. ...to engage in enough physical activity?	1	2	3	4	5
d. ...to maintain a healthy body weight?	1	2	3	4	5
e. ...to not smoke?	1	2	3	4	5

2. **Baseline: Setting priorities - self-management**

   Choose the top two areas related to your current self-management that you would **most like** to improve, and that you plan to work on actively over the coming period? Rank these in order of relevance, where priority 1 is the most relevant, and priority 2 the next most relevant.

   Self-management in the area of...
   1. taking medication
   2. healthy eating
   3. sufficient physical activity
   4. healthy body weight
   5. not smoking
   6. other (please state):

   a. Priority 1: ____________________

   b. Priority 2: ____________________

3. <u>Follow-up measurement: Score for self-perceived progress - self-management</u>

For the following statements about your current self-management, please indicate whether you have managed more or less successfully than the last time to carry out or maintain the behavior in question, or whether your situation has remained the same. It is not a problem if you do not remember exactly what you entered the last time: a rough estimate will do.

SELF-MANAGEMENT<br>Compared to the last time I completed this questionnaire, I have managed less well / better ...	*Much less well*	*Less well*	*Slightly less well*	*Equally well*	*Slightly better*	*Better*	*Much better*
1. to always take my medication as prescribed.	-3	-2	-1	0	1	2	3
2. to eat healthily.	-3	-2	-1	0	1	2	3
3. to engage in enough physical activity.	-3	-2	-1	0	1	2	3
4. to maintain a healthy body weight.	-3	-2	-1	0	1	2	3
5. to not smoke.	-3	-2	-1	0	1	2	3

4. <u>Posttest measurement: Areas actively worked on - self-management</u>

Have you tried to improve anything in any of these areas over the recent period?

If so, please choose up to two areas below that you have actively tried to improve recently. Rank these two areas, where number 1 is the area you have worked on most, and number 2 the area you have worked on slightly less.

If you have not actively worked on any of these areas, choose "not applicable".

Self-management in the area of...

1. taking medication
2. healthy eating
3. sufficient physical activity
4. healthy body weight
5. not smoking
6. other (please state):
7. not applicable

Area 1: I worked on my self-management in the area of ____________________

Area 2: I worked on my self-management in the area of ____________________
