# Supplementary material for: The Personalized Priority and Progress Questionnaire (PPPQ): A personalized instrument for quality of life and self-management for use in clinical trials and practice
Source: Qual Life Res. 2023 May 12;32(10):2789–803. doi: 10.1007/s11136-023-03429-7 (PMC10474184; doi:10.1007/s11136-023-03429-7)
Supplement: Supplementary file 2 — Dutch version PPPQ (PDF 199 KB) [file 11136_2023_3429_MOESM2_ESM.pdf]

Personalized Priority and Progress Questionnaire (PPPQ)

Kwaliteit van leven

1. **<u>Baseline: Scoren huidig functioneren – algemeen</u>**
   Onderstaande vragen gaan over beperkingen die mensen **door hun nieraandoening** kunnen ervaren. Denk aan de afgelopen 2 weken. Geef aan in hoeverre u **beperkingen ervaart op de volgende gebieden**.

ALGEMEEN<br>In welke mate ervaart u beperkingen op het gebied van…	*Helemaal niet*	*Enigszins*	*Nogal*	*Veel*	*Heel erg veel*
a. …vermoeidheid of slaapproblemen?	1	2	3	4	5
b. …pijn?	1	2	3	4	5
c. …jeuk?	1	2	3	4	5
d. …spanning, angst of bezorgdheid?	1 1	2 2	3	4	5
e. …een sombere of neerslachtige stemming?	1	2	3	4	5
f. …uw sociale omgeving (bijv. communicatie over uw behoeften of wensen, het vragen of ontvangen van steun)?	1	2	3	4	5
g. …dagelijkse activiteiten (bijv. bij werk, hobby's of sociale activiteiten)?	1	2	3	4	5
h. …afhankelijkheid van anderen?	1	2	3	4	5

2. **<u>Baseline: Prioriteiten stellen – algemeen</u>**
   Maak een top 2 van gebieden die te maken hebben met uw ervaren beperkingen, die u het **liefst** zou willen verbeteren en waarop u zich actief wilt inzetten de komende tijd. Hierbij vindt u prioriteit 1 het meest relevant en prioriteit 2 iets minder relevant van de twee gebieden die u kiest.

   Beperkingen op het gebied van…
   1. vermoeidheid of slaapproblemen
   2. pijn
   3. jeuk
   4. spanning, angst of bezorgdheid
   5. sombere of neerslachtige stemming
   6. sociale omgeving
   7. dagelijkse activiteiten
   8. afhankelijkheid
   9. anders, namelijk …

   a. Prioriteit 1: ___________________

   b. Prioriteit 2: ___________________

3. Nameting: Scoren zelfwaargenomen verandering – algemeen

Onderstaande vragen gaan over beperkingen die mensen **door hun nieraandoening** kunnen ervaren. Geef voor onderstaande stellingen aan of u ten opzichte van de vorige keer dat u deze vragenlijsten invulde meer of minder **beperkingen** ervaart, of dat uw situatie gelijk gebleven is. Het is niet erg als u niet meer precies weet wat u toen heeft ingevuld, een globale inschatting is voldoende.

ALGEMEEN<br>In vergelijking met de vorige keer dat ik deze vragenlijst invulde ervaar ik meer/minder beperkingen op het gebied van…	*Veel meer*	*Meer*	*Een beetje meer*	*Gelijk gebleven*	*Een beetje minder*	*Minder*	*Veel minder*
a. vermoeidheid of slaapproblemen.	-3	-2	-1	0	1	2	3
b. pijn.	-3	-2	-1	0	1	2	3
c. jeuk.	-3	-2	-1	0	1	2	3
d. spanning, angst of bezorgdheid.	-3	-2	-1	0	1	2	3
e. een sombere of neerslachtige stemming.	-3	-2	-1	0	1	2	3
f. mijn sociale omgeving (bijv. communicatie over mijn behoeften of wensen, het vragen of ontvangen van steun).	-3	-2	-1	0	1	2	3
g. dagelijkse activiteiten (bijv. bij werk, hobby's of sociale activiteiten).	-3	-2	-1	0	1	2	3
h. afhankelijkheid van anderen.	-3	-2	-1	0	1	2	3

4. Nameting: Gebieden waar actief aan gewerkt is – algemeen

Heeft u in de afgelopen periode geprobeerd om iets te verbeteren in een van deze gebieden?

Zo ja, kies hieronder maximaal twee gebieden die u de afgelopen tijd actief hebt proberen te verbeteren. Maak een top 2, waarbij u het meest bezig bent geweest met nummer 1 en iets minder met nummer 2 van de twee gebieden die u kiest.

Als u aan geen van deze gebieden actief hebt gewerkt, kunt u de optie "niet van toepassing" invullen.

Beperkingen op het gebied van…

1. vermoeidheid of slaapproblemen
2. pijn
3. jeuk
4. spanning, angst of bezorgdheid
5. sombere of neerslachtige stemming
6. mijn sociale omgeving
7. dagelijkse activiteiten
8. afhankelijkheid
9. anders, namelijk …
10. niet van toepassing

Gebied 1: Ik heb gewerkt aan beperkingen op het gebied van ____________________

Gebied 2: Ik heb gewerkt aan beperkingen op het gebied van ____________________

Zelfmanagement

1. **<u>Baseline: Scoren huidig functioneren – zelfmanagement</u>**
   Onderstaande vragen gaan over zelfmanagement. Denk aan de afgelopen 2 weken. Geef aan in hoeverre het u lukt om gezond te leven op de volgende gebieden.

ZELFMANAGEMENT<br>In welke mate lukt het u…	*Helemaal niet*	*Enigszins*	*Redelijk goed*	*Goed*	*Heel erg goed*
a. …om uw medicijnen altijd volgens voorschrift te nemen?	1	2	3	4	5
b. …om gezond te eten?	1	2	3	4	5
c. …om voldoende te bewegen?	1	2	3	4	5
d. …om een gezond lichaamsgewicht aan te houden?	1	2	3	4	5
e. …om niet te roken?	1	2	3	4	5

2. **<u>Baseline: Prioriteiten stellen – zelfmanagement</u>**
   Maak een top 2 van gebieden die te maken hebben met uw huidige zelfmanagement, die u het **liefst** zou willen verbeteren en waarop u zich actief wilt inzetten de komende tijd. Hierbij vindt u prioriteit 1 het meest relevant en prioriteit 2 iets minder relevant van de twee gebieden die u kiest.

   Zelfmanagement op het gebied van…
   1. medicijnen nemen
   2. gezond eten
   3. voldoende bewegen
   4. gezond lichaamsgewicht
   5. niet roken
   6. anders, namelijk …

   a. Prioriteit 1: ____________________

   b. Prioriteit 2: ____________________

3. <u>Nameting: Scoren zelfwaargenomen verandering – zelfmanagement</u>

Geef voor onderstaande stellingen over uw huidige zelfmanagement aan of het uitvoeren of volhouden ervan u ten opzichte van de vorige keer minder goed of beter lukt, of dat uw situatie gelijk gebleven is. Het is niet erg als u niet meer precies weet wat u toen heeft ingevuld, een globale inschatting is voldoende.

ZELFMANAGEMENT<br>In vergelijking met de vorige keer dat ik deze vragenlijst invulde lukt het mij minder goed/beter om…	*Veel minder goed*	*Minder goed*	*Een beetje minder goed*	*Even goed*	*Een beetje beter*	*Beter*	*Veel beter*
1. mijn medicijnen altijd volgens voorschrift te nemen.	-3	-2	-1	0	1	2	3
2. gezond te eten.	-3	-2	-1	0	1	2	3
3. voldoende te bewegen.	-3	-2	-1	0	1	2	3
4. een gezond lichaamsgewicht aan te houden.	-3	-2	-1	0	1	2	3
5. niet te roken.	-3	-2	-1	0	1	2	3

4. <u>Nameting: Gebieden waar actief aan gewerkt is – zelfmanagement</u>

Heeft u in de afgelopen periode geprobeerd om iets te verbeteren in een van deze gebieden?

Zo ja, kies hieronder maximaal twee gebieden die u de afgelopen tijd actief hebt proberen te verbeteren. Maak een top 2, waarbij u het meest bezig bent geweest met nummer 1 en iets minder met nummer 2 van de twee gebieden die u kiest.

Als u aan geen van deze gebieden actief hebt gewerkt, kunt u de optie 'niet van toepassing' invullen.

Zelfmanagement op het gebied van…

1. medicijnen nemen
2. gezond eten
3. voldoende bewegen
4. gezond lichaamsgewicht
5. niet roken
6. anders, namelijk …
7. niet van toepassing

Gebied 1: Ik heb gewerkt aan mijn zelfmanagement op het gebied van ____________________

Gebied 2: Ik heb gewerkt aan mijn zelfmanagement op het gebied van ____________________
