# Supplementary material for: The Personalized Priority and Progress Questionnaire (PPPQ): A personalized instrument for quality of life and self-management for use in clinical trials and practice
Source: Qual Life Res. 2023 May 12;32(10):2789–803. doi: 10.1007/s11136-023-03429-7 (PMC10474184; doi:10.1007/s11136-023-03429-7)
Supplement: Supplementary file 3 — Intercorrelations PPPQ items (PDF 135 KB) [file 11136_2023_3429_MOESM3_ESM.pdf]

Exploratory intercorrelations PPPQ items

QoL items

The results of the exploratory intercorrelation analyses between the PPPQ QoL items as measured in the CKD and dialysis sample are shown in Table S1. Regarding the baseline items as measured in the CKD sample, moderate to large correlations were found between the fatigue item and the items on pain, itch, depression, and daily activities; between the pain and daily activities items; between the anxiety item and the items on depression, social environment, and daily activities; between the depression and social environment items; and between the social environment and daily activities items; with correlations varying from $r = .32$ (fatigue and depression) to $r = .65$ (anxiety and depression). Thus, these correlations indicated that experienced limitations in these QoL areas were associated with limitations in other QoL areas. Between the other items, insignificant or small correlations were found (r-values $\leq .27$). In the dialysis sample, moderate to large correlations were found between the fatigue item and the items on anxiety, depression, social environment, and dependency; between the itch and the social environment items; and between the depression item and the items on social environment and daily activities; with correlations varying from $r = .30$ (itch and social environment) to $r = .71$ (fatigue and social environment). Between the other baseline items, insignificant to small correlations were found (r-values $\leq .29$).

Regarding the progress items as measured in the CKD sample, moderate to large correlations were found between all of the progress items of the PPPQ, with correlations varying from $r = .38$ (fatigue and itch) to $r = .78$ (social environment and daily activities). Thus, these correlations indicated that progress in one QoL area is generally associated with progress in other QoL areas. In the dialysis sample, moderate to large correlations were found between the fatigue item and the items on pain, anxiety, depression, daily activities, and dependency; between the pain item and the items on itch and daily activities; between the itch and daily activities items; between the anxiety item and the items on depression, social environment, daily activities, and dependency; between the depression item and the items on daily activities and dependency; and between the social environment and daily activities items; with correlations varying from $r = .30$ (anxiety and social environment) to $r = .98$ (anxiety and depression). Between the other progress items, non-existent or small correlations were found (r-values $\leq .29$).

Self-management items

The results of the exploratory intercorrelation analyses between the PPPQ self-management items as measured in the CKD sample are shown in Table S2. Regarding the baseline items, moderate correlations were found between the healthy diet and weight maintenance items ($r = .45$) and between the physical activity and weight maintenance items ($r = .46$). Between the other baseline items insignificant or small correlations were found (r-values $\leq .28$). With regard to the progress items, large

correlations were found between the healthy diet item and the items on physical activity ($r = .64$) and weight maintenance ($r = .65$) and between the physical activity and weight maintenance items ($r = .61$). A moderate correlation was found between the medication adherence and non-smoking items ($r = .42$) Between the other progress items insignificant or small correlations were found (r-values $\leq .28$).

Table S1. Intercorrelations between the Quality of Life items of the Personalized Priority and Progress Questionnaire (PPPQ)

	CKD patients (*N* = 121)							Dialysis patients (*N* = 22)							
Baseline items	1.	2.	3.	4.	5.	6.	7.	1.	2.	3.	4.	5.	6.	7.	8.
1. Fatigue	-							-							
2. Pain	.37**	-						.08	-						
3. Itch	.32**	.17	-					.009	.17	-					
4. Anxiety	.27**	.12	.15	-				.51*	.13	-.07	-				
5. Depression	.33**	.07	.10	.65**	-			.43*	.11	.27	.23	-			
6. Social environment	.22*	.23*	.26**	.57**	.37**	-		.71*	.25	.30	.09	.49*	-		
7. Daily activities	.45**	.34**	.16	.33**	.26**	.43**	-	.18	.29	.05	-.22	.38	.17	-	
8. Dependency	-	-	-	-	-	-	-	-.33	.15	.16	-.25	-.03	.10	.10	-
Progress items															
1. Fatigue	-							-							
2. Pain	.48**	-						.32	-						
3. Itch	.38**	.46**	-					.13	.61**	-					
4. Anxiety	.56**	.49**	.59**	-				.64**	.11	.18	-				
5. Depression	.43**	.50**	.60**	.79**	-			.65**	.10	.24	.98**	-			
6. Social environment	.49**	.40**	.41**	.53**	.51**	-		.19	.09	.13	.30	.27	-		
7. Daily activities	.54**	.43**	.42**	.40**	.45**	.78**	-	.32	.60**	.41	.31	.31	.36	-	
8. Dependency	-	-	-	-	-	-	-	.42	.28	.29	.45*	.44*	.10	.12	-

**p*<.05; ***p*<.01. Abbreviations: CKD, chronic kidney disease.

Table S2. Intercorrelations between self-management items of the Personalized Priority and Progress Questionnaire (PPPQ) in a CKD sample (*N* = 121)

Baseline items	1.	2.	3.	4.	5.
1. Medication adherence	-				
2. Healthy diet	.26**	-			
3. Physical activity	-.01	.29**	-		
4. Weight maintenance	-.12	.45**	.46**	-	
5. Non-smoking	.28**	-.08	-.09	-.03	-
Progress items					
1. Medication adherence	-				
2. Healthy diet	.28**	-			
3. Physical activity	.20*	.64**	-		
4. Weight maintenance	.21*	.65**	.61**	-	
5. Non-smoking	.42**	.09	.04	.06	-

*$p<.05$; **$p<.01$. Abbreviation: CKD, chronic kidney disease.
